# Recipient Vessel Selection in Head and Neck Reconstruction

**Published:** 2017-12-22

**Authors:** Andrea Hiller, Jared Davis, Steven Schulz, Josh Henderson, B. J. Wilhelmi

**Affiliations:** ^a^School of Medicine, University of Louisville, Ky; ^b^Division of Plastic and Reconstructive Surgery, Department of Surgery, University of Louisville, Louisville, Ky

**Keywords:** recipient vessels, head and neck reconstruction, external carotid artery, vessel caliber, Poiseuille's law

## Abstract

**Objective:** Recipient vessel caliber may be the single most important variable for flow to free tissue transfer. We performed cadaveric dissection of the external carotid artery and its branches to analyze average diameter in order to determine an algorithm for recipient vessel selection in head and neck reconstruction. **Methods:** The external carotid artery and branches were exposed on 3 lightly embalmed male human cadavers, aged 82 to 85 years. Each vessel was dissected, and luminal diameters were recorded with calipers. **Results:** The proximal ECA had the greatest average diameter (4 ± 0.6 mm) and potential flow; followed by distal ECA (2.85 ± 0.4 mm) facial (2.0 ± 0.6 mm), lingual (1.65 ± 0.6 mm), superior thyroid (1 ± 0.3  mm), and superficial temporal (0.85 ± 0.4 mm). There was a trend towards size variation between sides of the same cadaver. **Conclusion:** The external carotid artery has the greatest internal diameter and potential blood flow. It should be considered, when feasible, especially for defects of the upper third of the head. For defects of the lower third, the facial artery and the lingual artery should be utilized before the smaller diameter superior thyroid artery. Vessel selection is more challenging in the setting of radiation therapy, complex trauma, and prior neck surgery. In these settings, it is useful to have knowledge of the vascular anatomy and an objective algorithm for recipient vessel selection.

Approximately 390,000 new cases of head and neck cancer are diagnosed annually worldwide.^1^ The mainstay of treatment for early and locoregionally advanced disease is surgery and radiotherapy.^2^ For advanced disease requiring complex or composite reconstruction, microvascular free tissue transfer has become the standard of care.^3^ Indications for flap reconstruction are expanding due to increased surgical experience, advances in microvascular technique, and broadened flap selection. There is also reduced morbidity for those undergoing such procedures.^4^^-^8 Success rates of 98% to 99% have been reported by most large free flap series.^9^^-^12 The guiding principles are to restore anatomy, function, and favorable cosmesis.^5^ One underestimated factor to successful free tissue transfer is recipient vessel selection.^8^^,^^13^

The most commonly used recipient arteries are branches of the external carotid system.^13^^-^15 Recipient vessel selection can be impacted by many factors, including defect location, quality of the available recipient vessels, pedicle length, and anastomotic method.^14^^,^^16^ Each one of these can have a substantial impact on the operation and outcome.^14^ Typically, the external carotid artery (ECA) gives off 6 branches before terminating as the maxillary and superficial temporal arteries. These include 3 anterior branches (superior thyroid, lingual, and facial), 1 medial branch (ascending pharyngeal), and 2 posterior branches (occipital and posterior auricular).^17^^-^19 The anterior branches are often preferred because of favorable orientation; however, there are multiple factors to consider when selecting a recipient vessels.^15^ Ideally, the recipient artery should be disease free, of suitable length, and have a similar diameter to the donor vessel.^8^

Studies have shown that as recipient vessel diameter decreases, they may be less reliable.^9^^,^^20^ Vessel caliber effects flow exponentially, and higher flow is superior for patency and flap survival.^21^ Poiseuille's law describes the relationship of flow through a conduit (Fig 1). In vivo, vessel length and blood viscosity do not change much under normal physiologic conditions and can be considered constants. Although blood is considered a non-Newtonian fluid, the relationships described in the equation still hold true.^22^^,^^23^ Poiseuille's law shows that vessel radius is the most dominant variable affecting flow, as an increased radius yields an exponential increase in flow. Higher flow equates to increased flap circulation, improving flap survival.

The goal of this study was to analyze the external carotid system and formulate an algorithm for recipient vessel selection.

## METHODS

Under 3.2× and 4.5× loupe magnification, external carotid arteries and branches were exposed on 3 lightly embalmed human cadavers (all male) ranging in age from 82 to 85 years (mean = 84 years) (Fig 2). None had prior neck operations. Each side branch was cut 1 cm distal to its origin, and the internal diameter measured with a surgical caliper (Fig 3). The external carotid was measured proximally and distally. Numbers were rounded up to the nearest 0.5 mm. Digital images were recorded.

## RESULTS

The ECA and all branches were present in all 3 cadaveric specimens. No significant luminal atherosclerosis was found. The internal diameter of the proximal ECA, distal ECA, superior thyroid artery, lingual artery, facial artery, and superficial temporal artery (Fig 4) had average diameters of 4.0 ± 0.6, 2.9 ± 0.4, 1.0 ± 0.3, 1.7 ± 0.6, 2.0 ± 0.6, and 0.9 ± 0.4, respectively (Table 1). When comparing vessels of the same cadaver, there was an insignificant trend toward size variation between sides (Table 2).

## DISCUSSION

Microvascular free tissue transfer is a useful option in the armamentarium for reconstruction of head and neck defects. It is technically challenging, but success rates are high.^9^^-^12 This can be attributed to improved patient selection, flap choice, surgical technique, and postoperative management.^4^ Flap selection for appropriate characteristics, pedicle size, and length is important, but a detailed review of flap donor sites and pedicles is beyond the scope of this discussion. However, pedicle length and diameter influence recipient vessel selection and the need for vein grafting. Once the flap has been selected, one of the most critical decisions a surgeon can make is the proper selection of recipient vessels.^8^^,^^13^ This requires a strong background in cervical vascular anatomy.

**Figure 1:**
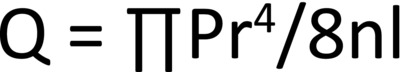
Poiseuille's law: *Q* = flow; η = viscosity, *P* = pressure gradient, *r* = radius, *L* = length.

**Figure 2:**
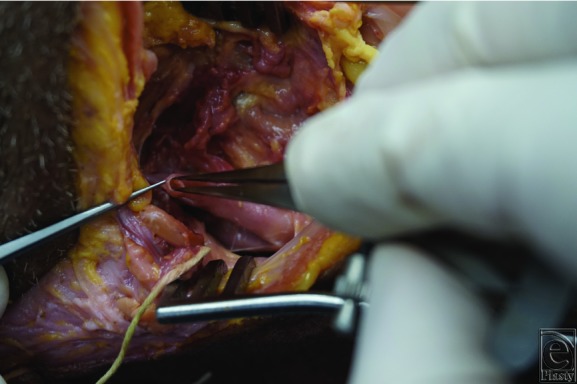
Vessel diameter measurement with a caliper.

**Figure 3:**
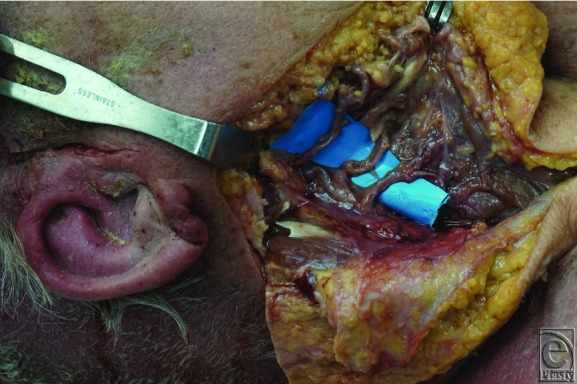
Dissected branches of the external carotid artery: superior thyroid, lingual, and facial.

**Figure 4:**
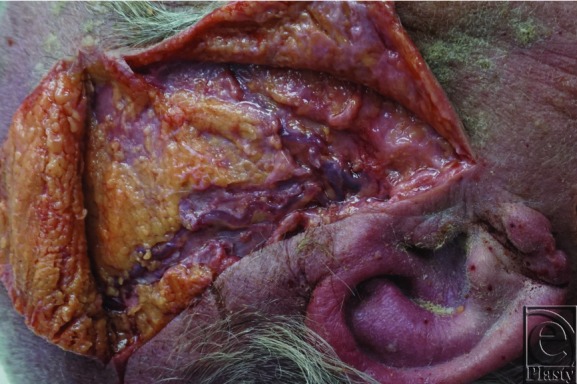
The superficial temporal artery, commonly used for defects in the upper third of the head.

The physiologic relationship between vessel size and blood flow is described by Poiseuille's equation (Fig 1). The dominant influence of radius on flow is seen in this relationship and can aid in understanding of how physiologic and pathologic changes can affect perfusion pressure and flow. For example, if the radius decreases by one half, the flow will be reduced 16-fold. The smaller vessel has 6% of the flow of the larger.^23^ Accordingly, our algorithm is based on vessel size and location.

The ECA had the largest diameter and therefore has the greatest potential blood flow. It should be considered whenever practical, especially for defects in the upper third of the head. The superficial temporal artery can also be considered but should be the second choice due to its significantly smaller diameter. For defects in the lower third of the face, the facial artery is preferred, followed by lingual and superior thyroid arteries. There have been several algorithms for recipient vessel selection published in literature. Other algorithms suggest superficial temporal vessels for upper-third defects and facial or the superior thyroid arteries for defects of lower two-thirds.^8^^,^^13^ These algorithms are not based on vessel diameter but based on several other features, including ease of dissection, historical reliability, and proximity to the defect. These are important factors, but size may be more important than credited.

As primary radiation therapy for malignancies of the head and neck and metachronous tumors becomes more commonplace, the choice for most appropriate recipient vessel can become obscured.^4^^,^^24^ Patients who have had prior treatment with any combination of chemotherapy, radiation therapy, or surgery pose a greater challenge.^15^ In some situations, vein grafting is required to access vessels that were outside the zone of injury.^25^^,^^26^ However, this requires longer operating times, increased number of microanastomoses, and lower patency.^15^ Although not examined in this study, the transverse cervical artery may be a useful alternative in the vessel-depleted neck. It has a reported average diameter of 2.65 mm,^15^ and multiple studies have concluded that it is reliable and accessible.^8^^,^^13^^,^^15^ Its venae comitantes drain into the external jugular system and can provide suitable outflow.

The complex vascular anatomy of the head and neck provides many options for recipient vessels. It is important for reconstructive surgeons to be familiar with vascular anatomy of the head and neck, especially the relative size of potential recipient arteries. Selection of venous outflow is also of significance but is beyond the scope of this study. Venae comitantes of the recipient artery, internal jugular, and external jugular are commonly used in an end-to-end or end-to-side fashion. Vein selection warrants further examination in future studies.

## Figures and Tables

**Table 1:** Average internal diameter of the ECA and selected branches*

Vessel	Diameter, mean ± SD
Proximal ECA	4.0 ± 0.6
Distal ECA	2.9 ± 0.4
Superior thyroid artery	1.0 ± 0.3
Lingual artery	1.7 ± 0.6
Facial artery	2.0 ± 0.6
Superficial temporal artery	0.9 ± 0.4

*Distance in millimeters. ECA indicates external carotid artery.

**Table 2:** Comparison of diameter of the ECA and selected branches*

Vessel	Cadaver 1	Cadaver 2	Cadaver 3	Average
Proximal ECA				
Right	4.0	3.5	4.5	4.0
Left	3.0	4.5	4.5	4.0
Distal ECA				
Right	3.0	3.0	3.0	3.0
Left	3.0	2.0	3.0	2.7
Superior thyroid artery				
Right	0.5	1.5	1.0	1.0
Left	1.0	1.0	1.0	1.0
Lingual artery				
Right	1.5	1.0	2.0	1.5
Left	2.5	1.0	2.0	1.8
Facial artery				
Right	2.5	1.5	3.0	2.3
Left	1.5	1.5	2.0	1.7
Superficial temporal artery				
Right	0.5	1.5	1.0	1.0
Left	0.5	0.5	1.0	0.7

*Distance in millimeters. ECA indicates external carotid artery.

## References

[1] Torre LA, Bray F, Siegel RL, Ferlay J, Lortet-Tieulent J, Jemal A (2015). Global cancer statistics, 2012. CA: Cancer J Clin.

[2] Rischin D, Ferris RL, Le Q-T (2015). Overview of advances in head and neck cancer. J Clin Oncol.

[3] Khouri RKR (1992). Free flap surgery. The second decade. Clin Plast Surg.

[4] Likhterov I, Urken M (2016). Mount Sinai Medical Center and their experience with unfavorable microsurgical head and neck reconstruction. Clin Plast Surg.

[5] Revenaugh PC, Fritz MA, Haffey TM, Seth R, Markey J, Knott P (2015). Minimizing morbidity in microvascular surgery: small-caliber anastomotic vessels and minimal access approaches. JAMA Facial Plast Surg.

[6] Urken MLM (1994). Microvascular free flaps in head and neck reconstruction. Report of 200 cases and review of complications. Arch Otolaryngol Head Neck Surg.

[7] Wong KK, Higgins KM, Enepekides DJ (2010). Microvascular reconstruction in the vessel-depleted neck. Curr Opin Otolaryngol Head Neck Surg.

[8] Yazar Sukru S (2007). Selection of recipient vessels in microsurgical free tissue reconstruction of head and neck defects. Microsurgery.

[9] Blackwell KE (1999). Unsurpassed reliability of free flaps for head and neck reconstruction. Arch Otolaryngol Head Neck Surg.

[10] Chalian AA, Anderson TD, Weinstein GS, Weber RS (2001). Internal jugular vein versus external jugular vein anastomosis: implications for successful free tissue transfer. Head Neck.

[11] Disa JJ, Pusic AL, Hidalgo DH, Cordeiro PG (2001). Simplifying microvascular head and neck reconstruction: a rational approach to donor site selection. Ann Plast Surg.

[12] Suh JD, Sercarz JA, Abemayor E (2004). Analysis of outcome and complications in 400 cases of microvascular head and neck reconstruction. Arch Otolaryngol Head Neck Surg.

[13] Chia HL, Wong CH, Tan BK, Tan KC, Ong YS (2011). An algorithm for recipient vessel selection in microsurgical head and neck reconstruction. J Reconstr Microsurg.

[14] Tan BK, Wong CH, Chen HC (2010). Anatomic variations in head and neck reconstruction. Semin Plast Surg.

[15] Tessler Oren O (2017). Transverse cervical artery: consistent anatomical landmarks and clinical experience with its use as a recipient artery in complex head and neck reconstruction. Plast Reconstr Surg.

[16] Nahabedian MY, Singh N, Deune EG, Silverman R, Tufaro AP (2004). Recipient vessel analysis for microvascular reconstruction of the head and neck. Ann Plast Surg.

[17] McMinn R (1990). Last's Anatomy: Regional and Applied.

[18] Cormack G, Lamberty B (1994). The Arterial Anatomy of Skin Flaps.

[19] Anderson J (1978). Grant's Atlas of Anatomy.

[20] Khouri RKR (1992). Avoiding free flap failure. Clin Plast Surg.

[21] Hanasono Matthew MM (2009). Changes in blood velocity following microvascular free tissue transfer. J Reconstr Microsurg.

[22] Lorenzetti F, Suominen S, Tukiainen E (2001). Evaluation of blood flow in free microvascular flaps. J Reconstr Microsurg.

[23] Klabunde RE (2012). Cardiovascular physiology concepts. Cardiovascular Physiology Concepts.

[24] Jacobson AS, Eloy JA, Park E, Roman B, Genden EM (2008). Vessel-depleted neck: techniques for achieving microvascular reconstruction. Head Neck.

[25] Jones NF, Johnson JT, Shestak KC, Myers EN, Swartz WM (1996). Microsurgical reconstruction of the head and neck: interdisciplinary collaboration between head and neck surgeons and plastic surgeons in 305 cases. Ann Plast Surg.

[26] Schusterman MA, Miller MJ, Reece GP, Kroll SS, Marchi M, Goepfert H (1994). A single center's experience with 308 free flaps for repair of head and neck cancer defects. Plast Reconstr Surg.

